# Lifelong SIRT-1 overexpression attenuates large artery stiffening with advancing age

**DOI:** 10.18632/aging.103322

**Published:** 2020-06-20

**Authors:** Daniel R. Machin, Yauling Auduong, Venkateswara R. Gogulamudi, Yu Liu, Md. Torikul Islam, Lisa A. Lesniewski, Anthony J. Donato

**Affiliations:** 1 University of Utah, Department of Internal Medicine, Salt Lake City, UT 84132, USA; 2 University of Utah, Department of Nutrition and Integrative Physiology, Salt Lake City, UT 84112, USA; 3 University of Utah, Department of Biochemistry, Salt Lake City, UT 84132, USA; 4 VA Salt Lake City, GRECC, Salt Lake City, UT 84148, USA

**Keywords:** arterial stiffness, aging, sirtuins

## Abstract

Advanced age is accompanied by aortic stiffening that is associated with decreased vascular expression of sirtuin-1 (SIRT-1). Interventions that increase SIRT-1 expression also lower age-related aortic stiffness. Therefore, we sought to determine if lifelong SIRT-1 overexpression would attenuate age-related aortic stiffening. Aortic pulse wave velocity (PWV) was assessed from 3-24 months in SIRT-1 transgenic overexpressing (SIRT^TG^) and wild-type (WT) mice. To determine the role of aortic structural changes on aortic stiffening, histological assessment of aortic wall characteristics was performed. Across the age range (3-24 mo), PWV was 8-17% lower in SIRT^TG^ vs. WT (P<0.05). Moreover, the slope of age-related aortic stiffening was lower in SIRT^TG^ vs. WT (2.1±0.2 vs. 3.8±0.3 cm/sec/mo, respectively). Aortic elastin decreased with advancing age in WT (P<0.05 old vs. young WT), but was maintained in SIRT^TG^ mice (P>0.05). There was an age-related increase in aortic collagen, advanced glycation end products, and calcification in WT (P<0.05 old vs. young WT). However, this did not occur in SIRT^TG^ (P>0.05). These findings indicate that lifelong SIRT-1 overexpression attenuates age-related aortic stiffening. These functional data are complemented by histological assessment, demonstrating that the deleterious changes to the aortic wall that normally occur with advancing age are prevented in SIRT^TG^ mice.

## INTRODUCTION

Cardiovascular diseases (CVDs) are the leading cause of death in the United States, and there is a progressive increase in the prevalence of CVD and CVD-related death with advancing age [1]. Among the many deleterious changes to the arterial phenotype that occur with advancing age, the stiffening of the large elastic arteries (i.e., aorta and carotid arteries) is one of the most important, as arterial stiffness is present in several CVD states, such as hypertension, stroke, and left ventricular hypertrophy [2]. Moreover, aortic stiffening is associated with an increased risk of cardiovascular events [3]. Thus, identifying treatments that can prevent or reverse age-related arterial stiffening is of paramount importance.

Sirtuins (SIRTs) are a family of nicotinamide adenine dinucleotide (NAD+)-dependent protein deacetylases [4] and ADP-ribosyltransferases that play a critical role in longevity [5]. Many of the physiological benefits that caloric restriction (CR) has on longevity are attributable to SIRTs, specifically, SIRT-1 [6, 7]. Indeed, genetic SIRT-1 deletion abolishes CR-mediated lifespan extension [8], whereas SIRT-1 overexpression recapitulates the CR phenotype [9]. Our group has demonstrated that declines in vascular SIRT-1 expression in advanced age are associated with age-related large artery stiffening [10]. Moreover, interventions that increase SIRT-1 activity, such as CR [10–12] and dietary supplementation with NAD^+^ intermediates [13, 14] are capable of preventing or lowering age-related aortic stiffness in mice [10–12, 14], as well as in humans [15]. Although these observations demonstrate the potential cardiovascular benefits of restoring SIRT-1 in the aged vasculature, these studies do not address the protective role that SIRT-1 might play as the vasculature ages.

Recent findings indicate that carotid artery stiffness is lower in old (~20-22 mo) SIRT-1 transgenic overexpressing (SIRT^TG^) mice, compared to age-matched wild-type (WT) mice [16], but we have observed a similar magnitude of difference in aortic stiffness between young SIRT^TG^ and WT mice (unpublished observations). Thus, it is unclear whether lifelong SIRT-1 overexpression attenuates the rate of arterial stiffening with age or that these animals simply start with lower arterial stiffness in youth. More importantly, no study has comprehensively examined aortic stiffness and structure across the lifespan in SIRT^TG^ mice. Accordingly, the primary aims of the present study were to measure aortic stiffness, as determined by pulse wave velocity (PWV), and structural changes at specific time points across the normal rodent lifespan (~24 months) in SIRT^TG^ and WT mice in an effort to gain a more complete understanding of the role of SIRT-1 in vascular aging. We hypothesized that in youth, aortic stiffness would be lower in SIRT^TG^ mice and that the rate of aortic stiffening would be attenuated in SIRT^TG^ mice. Moreover, we hypothesized that there would be an absence of the deleterious aortic structural changes in SIRT^TG^ mice that occur with advancing age.

## RESULTS

### Animal characteristics

Body mass in both young WT and SIRT^TG^ mice were lower than their middle-age counterparts (P<0.05) (Table 1), however, body mass in old SIRT^TG^ mice was similar to young (P>0.05), but lower than age-matched WT mice (P<0.05). In all age groups and in both genotypes, male mice had higher body mass than female mice (data not shown, P<0.05). Heart mass was greater in old WT and SIRT^TG^ mice compared to young mice (P<0.05). Both liver and spleen masses were greater in old WT and SIRT^TG^ mice compared to young mice (P<0.05). Lastly, perigonadal white adipose tissue (WAT) was unchanged across the lifespan and between genotypes (P>0.05).

**Table 1:** Animal characteristics.

	**WT**			**SIRT^TG^**		
	**Young**	**Middle-Age**	**Old**	**Young**	**Middle-Age**	**Old**
Male:female N	11:12	12:10	10:4	9:10	11:6	11:10
Age, mo	5.9±0.3	11.6±0.2^†^	22.9±0.5^†‡^	5.9±0.4	11.0±0.3^†^	23.2±0.2^†‡^
Body mass, g	27±1	31±1^†^	32±1^†^	27±1	32±1^†^	29±1*
Heart, mg	128±3	149±6^†^	192±8^†‡^	132±6	146±7	181±5^†‡^
Heart:body mass, mg/g	4.7±0.1	4.9±0.1	6.0±0.5^†‡^	4.8±0.2	4.8±0.2	6.3±0.2^†‡^
Quadriceps, mg	194±7	200±6	171±13^‡^	202±7	191±10	171±12^†^
Quadriceps:body mass, mg/g	6.9±0.2	6.6±0.2	5.8±0.4^†‡^	7.4±0.3	6.4±0.3^†^	5.6±0.3^†^
Gastrocnemius, mg	153±7	152±6	159±11	158±7	154±8	153±9
Gastrocnemius:body mass, mg/g	5.5±0.2	5.0±0.2	5.4±0.3	5.7±0.2	5.1±0.2	5.2±0.3
Soleus, mg	12±1	11±0	12±1	11±1	11±1	11±1
Soleus:body mass, mg/g	0.42±0.03	0.38±0.02	0.40±0.03	0.40±0.02	0.36±0.01	0.38±0.02
Liver, g	1.4±0.1	1.5±0.1	2.1±0.1^†‡^	1.4±0.1	1.6±0.1	2.0±0.1^†‡^
Spleen, mg	84±6	88±4	179±54^†‡^	71±4	74±3*	161±22^†‡^
Perigonadal WAT, mg	417±69	454±53	450±105	446±68	563±63	404±110

*P<0.05 vs. WT. ^†^P<0.05 vs. Young. ^‡^P<0.05 vs. Middle-Age. Data are mean±SEM.

White adipose tissue, WAT; Wild-type, WT; SIRT-1 transgenic overexpressing, SIRT^TG^.

### Increased aortic SIRT-1 mRNA expression across the lifespan with transgenic SIRT-1 overexpression

**Figure 1:**
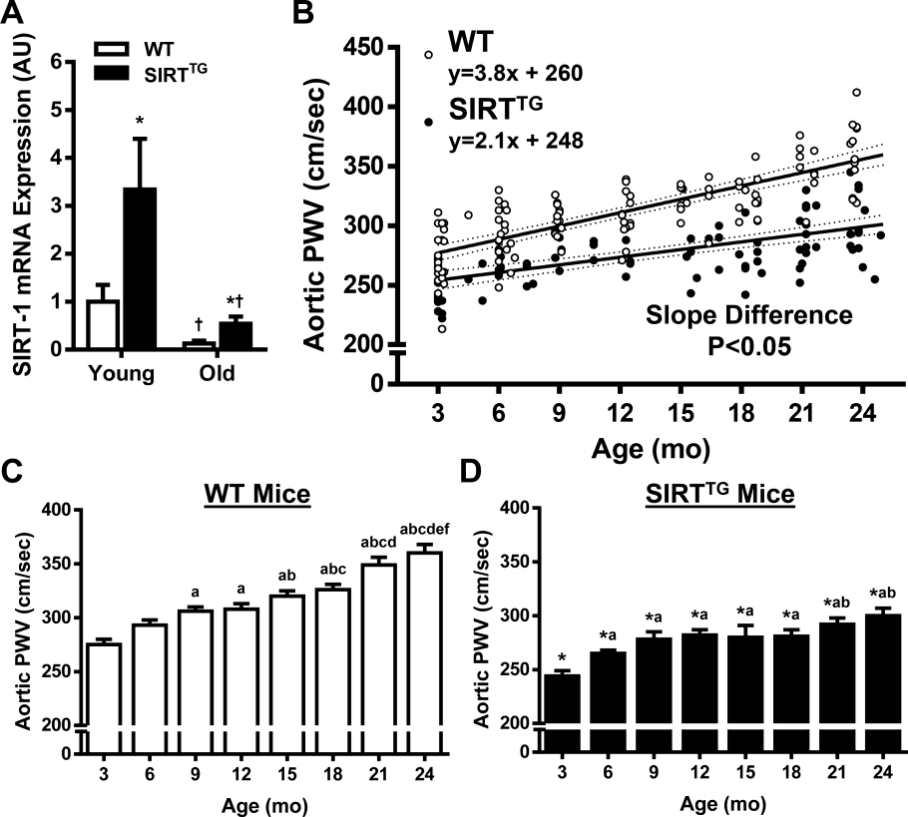
****Aortic SIRT-1 mRNA gene expression (**A**) in wild-type (WT) and SIRT-1 transgenic overexpressing (SIRT^TG^) mice (N=4-8/group) normalized to Young WT. Scatter plot (**B**) of aortic pulse wave velocity (PWV) with advancing age in WT and SIRT^TG^ mice. Separate regression lines are given for WT and SIRT^TG^ mice (dotted lines define 95% confidence intervals). Aortic PWV measured at 3 mo increments in WT (**C**) and SIRT^TG^ (**D**) mice (N=7-24/group). *P<0.05 vs. WT in same age group. ^†^P<0.05 vs. Young. a, b, c, d, e, f P<0.05 vs. 3, 6, 9, 12, 15, 18, 21 mo, respectively. Data are mean±SEM.

Young and old WT and SIRT^TG^ mice used for gene expression studies were 3.3±0.5, 3.1±0.6, 20.3±1.0, and 20.1±1.2 mo, respectively. Aortic gene expression of SIRT-1 was 3-fold higher in young SIRT^TG^ (3.3±0.5 mo), compared to young WT mice (Figure 1A; P<0.05). Advanced age resulted in a significant decrease in SIRT-1 mRNA expression in both old SIRT^TG^ and WT mice, compared to their young counterparts (P<0.05), still, SIRT-1 expression remained >3-fold higher in old SIRT^TG^ vs. WT mice (P<0.05).

### Age-related aortic stiffening is attenuated with lifelong SIRT-1 overexpression

A total of 206 aortic PWV measurements (WT: 111 measurements; SIRT^TG^: 95 measurements) were performed in 93 mice (WT: 24 male and 25 female; SIRT^TG^: 19 male and 25 female). There were no sex-related differences in PWV, as assessed by 3-factor ANOVA (age X genotype X sex). The slope of increase in aortic PWV with advancing age was greater in WT, compared to SIRT^TG^ mice (Figure 1B; 3.8±0.3 vs. 2.1±0.2 cm/sec, respectively, P<0.05). In each age group, aortic PWV was greater in WT, compared to SIRT^TG^ mice (Figure 1D; P<0.05). In WT mice, aortic PWV increases with age at most 3 mo increments (Figure 1C; P<0.05). Whereas aortic PWV in SIRT^TG^ mice increases from 3 to 9 mo old (Figure 1D; P<0.05), but does not rise significantly until 24 mo old. Anesthetized heart rate was similar between WT and SIRT^TG^ mice in all age groups (Table 2; P>0.05).

**Table 2:** Anesthetized heart rate.

	**Age, mo**							
	**3**	**6**	**9**	**12**	**15**	**18**	**21**	**24**
Heart rate, bpm								
WT	433±9	461±13	445±16	435±14	392±10^a^	431±17	468±11^e^	407±11^g^
SIRT^TG^	439±14	437±13	460±17	463±19	414±12	430±13	448±11	416±12

*P<0.05 vs. WT. ^a, b, c, d, e, f, g^ P<0.05 vs. 3, 6, 9, 12, 15, 18, 21 mo, respectively. Data are mean±SEM.

Wild-type, WT; SIRT-1 transgenic overexpressing, SIRT^TG^.

### Deleterious age-related structural changes to the aorta are prevented with lifelong SIRT-1 overexpression

**Figure 2:**
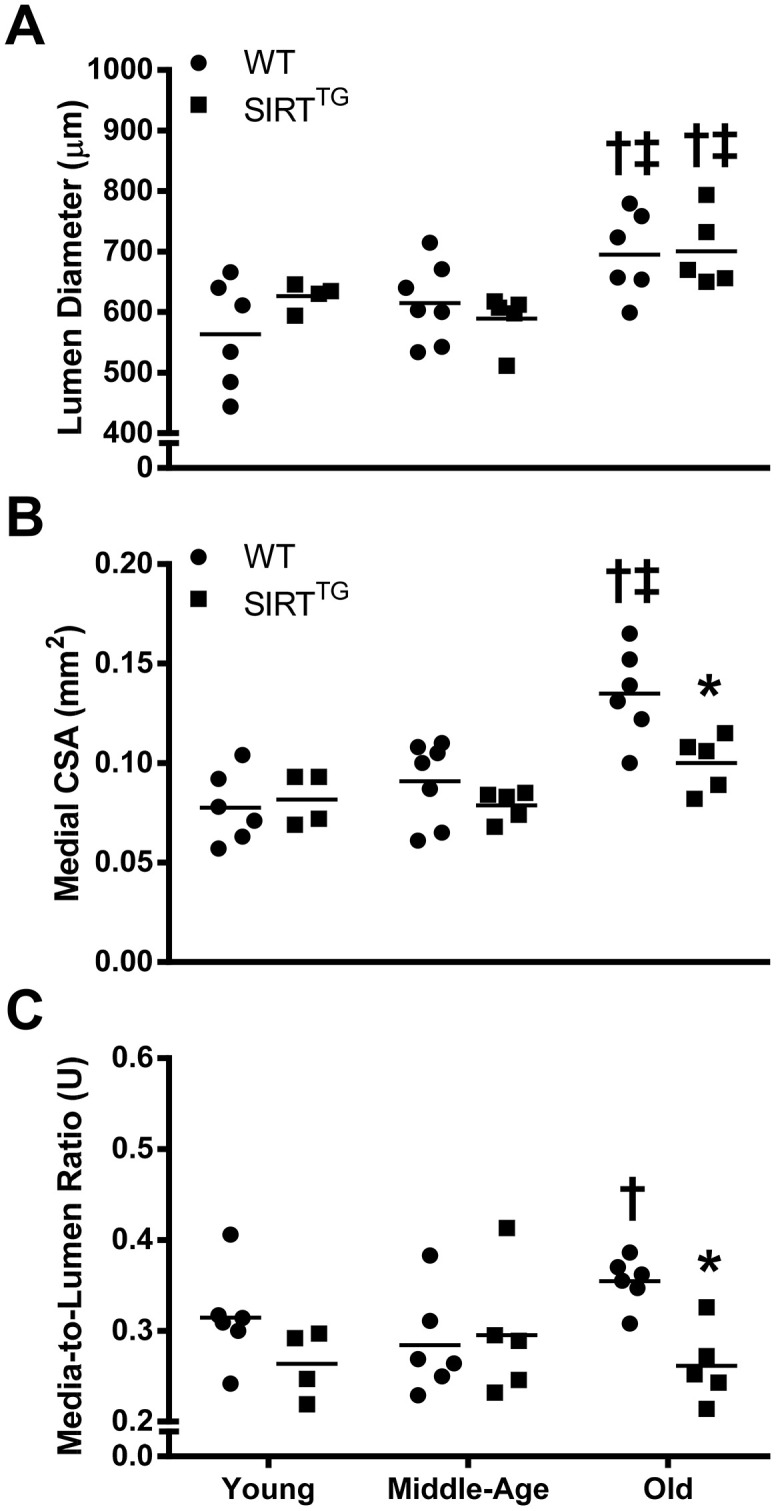
****Thoracic aorta lumen diameter (**A**), medial cross-sectional area (**B** [CSA]), and media-to-lumen ratio (**C**) in young, middle-age, and old wild-type (WT) and SIRT-1 transgenic overexpressing (SIRT^TG^) mice. *P<0.05 vs. WT in same age group. ^†^P<0.05 vs. Young. ^‡^P<0.05 vs. Middle-Age. Data are individual values and means.

In histological sections of thoracic aortas excised from young, middle-age, and old mice, lumen diameter was greater in both old WT and SIRT^TG^ mice compared with their young and middle-age counterparts (Figure 2A; P<0.05). However, medial cross-sectional area (Figure 2B) was greater in old WT, compared with young and middle-age WT, as well as old SIRT^TG^ mice (P<0.05). After normalizing lumen area (Figure 2C), there was a greater media-to-lumen ratio in old WT, compared with young WT and old SIRT^TG^ mice (P<0.05). Conversely, there were no differences in medial cross-sectional area or media-to-lumen ratio between age groups in SIRT^TG^ mice (Figure 2B, 2C; P>0.05).

**Figure 3:**
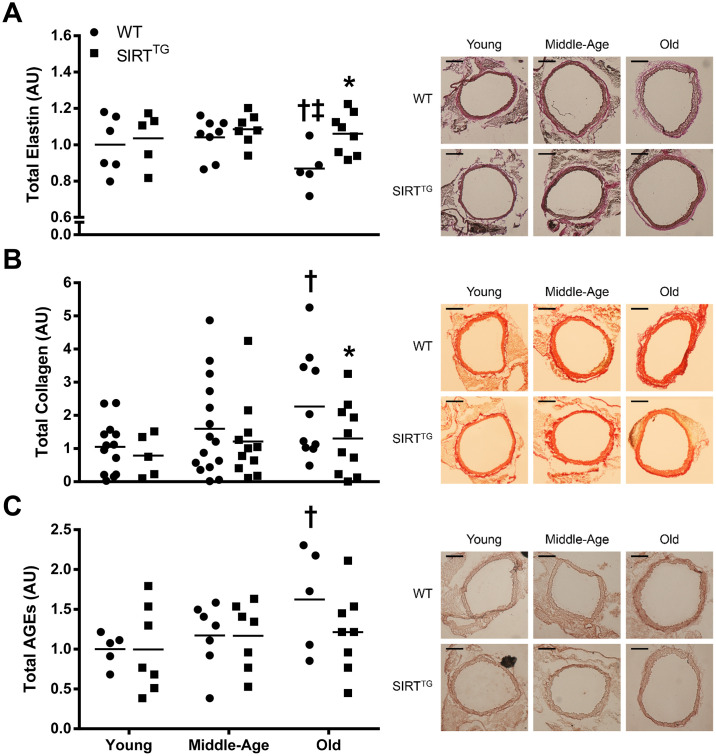
Total thoracic aortic elastin (**A**), collagen (**B**), and advanced glycation end products (**C** [AGEs]) content in young, middle-age, and old wild-type (WT) and SIRT-1 transgenic overexpressing (SIRT^TG^) mice. Figures are accompanied by representative images of elastin, collagen, and AGEs staining. Black scale bars are equal to 200 μm. *P<0.05 vs. WT. ^†^P<0.05 vs. Young. ^‡^P<0.05 vs. Middle-Age. Data are individual values and means normalized to Young WT.

**Figure 4:**
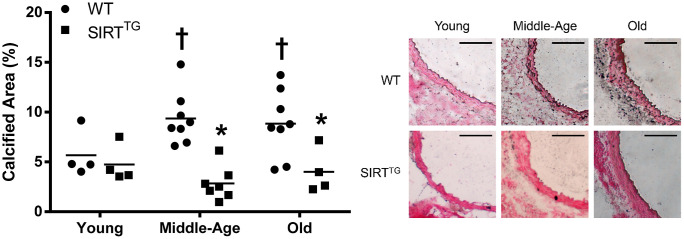
Calcified area relative to tunica media area in young, middle-age, and old wild-type (WT) and SIRT-1 transgenic overexpressing (SIRT^TG^) mice. Figure is accompanied by representative images. Black scale bars are equal to 100 μm. *P<0.05 vs. WT. ^†^P<0.05 vs. Young. Data are individual values and means.

Elastin content was lower in old WT, compared with young and middle-age WT, as well as with old SIRT^TG^ mice (Figure 3A; P<0.05). Aortic collagen content was higher in old WT, compared with young WT, as well as old SIRT^TG^ mice (Figure 3B; P<0.05). Although there was no difference in aortic advanced glycation end products (AGEs) intensity in any age group between WT and SIRT^TG^ mice, AGEs intensity was higher in old WT, compared with young WT mice (Figure 3C; P<0.05). Calcified area was higher in middle-age and old WT, compared to young WT, as well as compared with age-matched SIRT^TG^ mice (Figure 4; P<0.05). Lastly, there was no difference in elastin, collagen, AGEs, and calcified area between age groups in the SIRT^TG^ mice (Figures 3, 4; P>0.05).

### SIRT-1 overexpression augments aortic superoxide dismutase that remains elevated in advanced age

**Figure 5:**
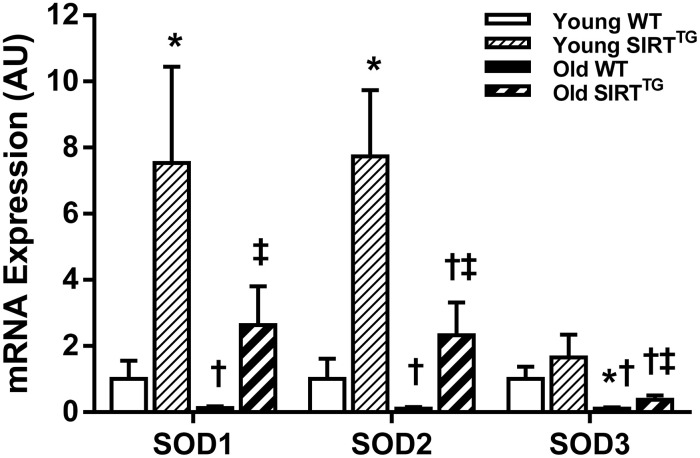
**Aortic SOD1, SOD2, and SOD3 mRNA gene expression in young and old wild-type (WT) and SIRT-1 transgenic overexpressing (SIRT^TG^) mice (N=5-7/group).** *P<0.05 vs. Young WT. †P<0.05 vs. Young SIRT^TG^. ‡P<0.05 vs. Old WT. Data are mean±SEM normalized to Young WT.

Aortic gene expression of superoxide dismutase (SOD) isoforms, SOD1, SOD2, and SOD3, trended toward being lower with advancing age, but were augmented by lifelong SIRT-1 overexpression (Figure 5). Specifically, we observed lower SOD3 gene expression (P<0.05) and a trend toward lower SOD1 and SOD2 gene expression (P=0.07-0.11) in old compared to young WT mice. Whereas, gene expression of all three SOD isoforms was lower in old compared to young SIRT^TG^ mice (P<0.05). We also observed higher SOD1 and SOD2 in young and old SIRT^TG^ mice compared with age-matched WT mice (P<0.05), while SOD3 was only higher in old SIRT^TG^ mice compared with age-matched WT mice (P<0.05).

## DISCUSSION

In the present study, we comprehensively examined aortic stiffness and structural changes across the normal rodent lifespan in WT and SIRT^TG^ mice. In comparison to WT, we observed a lower rate of age-related aortic stiffening in SIRT^TG^ mice. Additionally, the deleterious aortic structural changes that occurred in old WT mice, such as medial wall hypertrophy, reduced elastin, accumulation of collagen, AGEs, and aortic calcification were absent in old SIRT^TG^ mice. These findings indicate that lifelong SIRT-1 overexpression attenuates aortic stiffening and prevents many of the deleterious structural changes to the aorta that normally occur with advancing age.

### SIRT-1 overexpression and aortic stiffening

Aortic stiffening with advancing age accompanies decreased vascular SIRT-1 expression and activity [10]. Interestingly, elevations in SIRT-1 activity that are achieved via CR or dietary supplementation with NAD^+^ intermediates are capable of decreasing aortic stiffness in old mice [10–12, 14], as well as in older humans [15]. In the present study, we measured aortic stiffness in WT and SIRT^TG^ mice at ~3-month intervals from 3-24 mo old. In addition to having lower aortic stiffness in youth, the rate of aortic stiffening across the lifespan in SIRT^TG^ mice was nearly half that of WT mice. Previously, whole-body SIRT-1 transgenic overexpression resulted in lower carotid artery stiffness in old mice, compared to age-matched WT mice [16]. However, the findings from that study provide only a snapshot of arterial stiffness at one time point in advanced age. Whereas in the present study, through our comprehensive examination aortic stiffness and structure across the lifespan, provides a more complete understanding on the role of SIRT-1 in vascular aging.

Despite the aforementioned benefits of SIRT-1 overexpression, we still observed an increase in aortic stiffness between some age groups in SIRT^TG^ mice, although the majority of aortic stiffening in SIRT^TG^ mice occurred during development. Increases in aortic stiffness during development are likely due to increases in body length, as we observed a similar increase in PWV during youth in WT mice. Thus, increases in aortic stiffness during development from 3-9 mo are likely trivial to vascular health. Still, age-related aortic stiffening in SIRT^TG^ mice did occur at 21-24 mo, which could be due to declines in aortic SIRT-1 gene expression in advanced age. However, despite a decrease in aortic SIRT-1 expression in old SIRT^TG^ mice, SIRT-1 expression remained at least 3-fold higher than that of age-matched WT mice, but was 50% that of young WT mice. Increases in SIRT-1 activity via CR or dietary supplementation with NAD^+^ reverse aortic stiffness in old mice [10–12, 14]. Considering the age-related decline in aortic SIRT-1 expression in old SIRT^TG^ mice, it is possible that either of these interventions could prevent aortic stiffening that occurred beyond development in these mice. Although we cannot pinpoint a direct mechanism in the present study, it is clear that lifelong SIRT-1 overexpression results in a healthier vascular phenotype in advanced age. It is important to note that the benefits of increased SIRT-1 activation do not need to be lifelong in order to exert vasoprotective effects, as reductions in aortic stiffness in humans and mice can occur in the short-term by increasing SIRT-1 activity in advanced age [10–14]. Future studies are warranted to determine if lifelong maintenance of aortic SIRT-1 at youthful levels is capable of preventing the entirety of age-related aortic stiffening that occurs beyond development.

### SIRT-1 overexpression and aortic structure

Unlike aortic stiffness, there was no difference in any aortic structural variable measured between young WT and SIRT^TG^ mice. Although with advancing age both groups demonstrated an increase in aortic lumen diameter, only WT mice developed deleterious structural changes to their aortic wall. In WT mice, there was an increase in aortic medial cross-sectional area with advancing age, but this did not occur in SIRT^TG^ mice. Because increased medial cross-sectional area may be a consequence of the normal age-related widening of the aortic lumen, we normalized medial cross-sectional area to lumen area. However, media-to-lumen ratio was also elevated in old WT compared to both young WT and old SIRT^TG^ mice. Resistance to medial hypertrophy/hyperplasia has also been demonstrated in cultured rat aortic vascular smooth cells that overexpress SIRT-1 [17]. Thus, in the context of aging, it is likely that SIRT-1 overexpression provides a similar resistance to age-related medial hypertrophy/hyperplasia. Although lumen diameter is greater in advancing age in both SIRT^TG^ and WT mice, the increased lumen diameter in SIRT^TG^ mice does not appear to be deleterious because medial hypertrophy does not accompany it.

In addition to aortic wall dimensions, we also examined the structural components of the aorta. In WT mice, there was an age-related reduction in aortic elastin content with a corresponding increase in collagen content. Unlike WT mice, aortic elastin and collagen content were unchanged with advancing age in SIRT^TG^ mice. Changes in elastin and collagen content are a common alteration in aged arteries [18]. The lack of change in aortic elastin and collagen content with advancing age indicates that SIRT-1 overexpression provides resistance to these deleterious structural changes of aging. It is possible that SIRT-1 exerts these beneficial effects by direct acetylation of collagen and/or elastin, but to the best of our knowledge, this remains unknown. Indeed, SIRT-1 is a positive regulator of tissue inhibitor of matrix metalloproteinase-1 (TIMP-1) and a negative regulator of matrix metalloproteinase-9 (MMP-9) [19, 20], which might provide insight in the ability of SIRT-1 to maintain aortic elastin content in advanced age [21, 22]. Moreover, increased arterial SIRT-1 activity via dietary supplementation with NAD^+^ intermediates that lowers age-related aortic stiffness also normalizes aortic elastin and collagen content in old mice [14]. The present study is supportive of previous work, indicating that elevated SIRT-1 expression attenuates aortic stiffening and changes in aortic elastin and collagen content in advanced age.

In addition to maintaining a youthful elastin and collagen content, accumulation of AGEs and calcified area in the aorta were also unchanged with advancing age in SIRT^TG^ mice. Conversely, aortic AGEs and calcium content were both elevated with aging in old WT mice. Direct activation of SIRT-1 via the small molecule, SRT-1720, preserves glucose tolerance in aged rodents, preventing the accumulation of AGEs in SIRT^TG^ mice via maintenance of normal glucose tolerance in advanced age [23]. In addition to preventing structural changes, maintaining a properly functioning vascular endothelium may also play a pivotal role. SIRT-1 plays a direct role in modulating nitric oxide (NO) and endothelial function via deacetylation and subsequent activation of endothelial NO synthase (eNOS) [24, 25]. Moreover, transgenic whole-body SIRT-1 overexpression prevents the age-related decline in endothelium-dependent vasodilation [16]. Thus, maintaining a properly functioning endothelium in the aged vasculature may also be a benefit of lifelong SIRT-1 overexpression that could impart vasoprotective benefits leading to attenuated aortic stiffness.

Lastly, as mentioned previously, it should be noted that there was a slight, but significant increase in aortic stiffness in old compared to young SIRT^TG^ mice. Although old SIRT^TG^ mice had elevated aortic stiffness, their PWV values were similar to that of young WT mice. Thus, these data indicate that the deleterious structural changes to the aorta we observed in older WT mice may have been driven by aortic stiffness after it surpassed a critical threshold in midlife or later. Notably, these structural changes to the aorta were prevented in SIRT^TG^ mice by maintaining a youthful aortic stiffness across the lifespan. Taken together, these data indicate that lifelong SIRT-1 overexpression prevents deleterious structural changes to the aged aorta, possibly through attenuations in age-related aortic stiffening.

### Experimental considerations

This study is not without limitations. It is well established that SIRT-1 activity increases to elevations in intracellular NAD+ that occur in response to energy/nutrient stresses, such as caloric restriction [26] and exercise [27]. Therefore, higher aortic SIRT-1 gene expression in SIRT^TG^ permits a greater increase in SIRT-1 activity in response to a given change in NAD+ levels. However, we did not measure NAD+ or make indirect measures of SIRT-1 activation, thus, we are unable to confirm this assumption. Furthermore, we did not measure food consumption or spontaneous cage activity in this study. Therefore, it is possible that the attenuated slope of age-related aortic stiffening may have been due to lower food consumption and/or higher activity levels in SIRT^TG^ mice. Still, we see no differences in body mass or heart and skeletal muscle masses normalized to body mass between genotypes, suggesting that it is unlikely there were differences in food consumption or activity level that contributed to a lower rate of aortic stiffening in SIRT^TG^ mice.

### Summary and future directions

These findings indicate that lifelong SIRT-1 overexpression results in lower aortic stiffness at any age across the lifespan, but also slows the rate of aortic stiffening occuring with advancing age. These functional data are complemented by histological assessement of aorta structural characteristics, demonstrating that the deleterious changes to the aorta that normally occur with advancing age, such as medial wall hypertrophy, reduced elastin, accumulation of collagen and AGEs, and aortic calficification are all prevented in mice that are afforded lifelong SIRT-1 overexpression. Although it is unknown if SIRT-1 directly effects any of these processes, we also demonstrated elevated aortic SOD1, SOD2, and SOD3 gene expression in SIRT^TG^ mice that suggests SIRT-1 augments antioxidant capacity, which likely attenuates fibrosis, indirectly influencing these processes. Future studies are warranted to determine specific mechanisms by which SIRT-1 exerts its anti-stiffening effects and the use of tissue-specific models of SIRT-1 overexpression or deletion may provide greater mechanistic insight into that area. In summary, these findings indicate that lifelong SIRT-1 overexpression negates many of the deleterious alterations to the aorta that occur with advancing age. Thus, the use of SIRT-1 activators to prevent or reverse the age-related aortic stiffness may be a more viable approach compared to other lifestyle interventions, such as CR.

## MATERIALS AND METHODS

### Animals

Male and female SIRT^TG^ and WT littermate control mice on a C57BL/6 background were generated from breeding colonies at the Veteran’s Affairs Medical Center-Salt Lake City (VAMC-SLC) [29]. Animals used in this study were housed in the animal care facility at the VAMC-SLC on a 12:12 light:dark cycle and fed standard rodent chow (Envigo, Teklad Diet #8604). Food and water were supplied *ad libitum*.

### Quantitative PCR

mRNA expression of SIRT-1 was measured in the aorta of young and old mice by quantitative PCR. Briefly, RNA isolated from aortic tissue was used to synthesize cDNA via QuantiTect Reverse Transcription kit (Qiagen, Inc., Valencia, CA, USA). Quantitative PCR was performed using RT^2^ SYBR^®^ Green quantitative PCR Mastermix (Qiagen, Inc.). Fold change in mRNA expression was calculated as the fold difference in expression of target mRNA to 18s rRNA $2^{-(\text{target}\;{\text{C}}_{\text{T}}-18\text{s}\;{\text{C}}_{\text{T}})}$ and normalized to young values. 18s primer sequences: forward: TAGAGGGACAAGTGGCGTTC; reverse: CGCTGAGCCAGTCAGTGT. SIRT-1 primer sequences: forward: GATTGGCACAGATCCTCGAA; reverse: GTCTACAGCAAGGCGAGCATA. SOD1 primer sequences: forward: AACCAGTTGTGTTGTCAGGAC; reverse: CCACCATGTTTCTTAGAGTGAGG. SOD2 primer sequences: forward: CAGACCTGCCTACGACTATGG; reverse: CTCGGTGGCGTTGAGATTGTT. SOD3 primer sequences: forward: CCTTCTTGTTCTACGGCTTGC; reverse: TCGCCTATCTTCTCAACCAGG.

### Aortic stiffness

Aortic PWV was determined *in vivo* at ~3-month increments over the normal lifespan of C57BL/6 mice (~24 months; 206 measurements in total). Briefly, mice were anesthetized with isoflurane (2-3%) in 100% oxygen at 2 L/min flow rate and placed in the supine position on a heated platform (37° C). Blood velocity waveforms at the transverse aortic arch and at the abdominal aorta were measured simultaneously with two 20-MHz Doppler probes (Indus Instruments, Webster, TX, USA) and recorded using WinDAQ Pro + software (DataQ Instruments, Akron, OH, USA). After blood velocities were collected, a precise measurement of the traveled distance between the Doppler probes was recorded using a scientific caliper. The transit time between Doppler sites was determined using the foot-to-foot method with WinDAQ Waveform Browser (DataQ Instruments). Aortic PWV was calculated as the traveled distance divided by the transit time.

### Aortic histology

Young (5-7 months), middle-age (12-13 months), and old (23-24 months) SIRT^TG^ and WT mice were sacrificed by exsanguination via cardiac puncture under isoflurane anesthesia. Thoracic aortas were quickly excised and placed in cold (4°C) physiological salt solution. For each mouse, an 2-3 mm aortic ring with perivascular tissue intact was excised from the thoracic aorta and embedded in Optimal Cutting Temperature medium. Aortic rings were sliced into 8-micron sections. Each mouse aorta had 3 to 4 sections per slide, which were averaged. For measures of medial cross-sectional area the lumen border and the outer medial border were traced in ImageJ and internal areas measured. These areas were used to calculate medial cross-sectional area and were calculated as the outer media border area minus the lumen area. Elastin was quantified by Verhoff’s Van Geison stain as percentage of the selected area, as described previously [30]. An 8-bit grayscale was used for densitometric quantification of elastin content with ImageJ. Collagen was quantified by picrosirius red stain as percentage of the selected area, as described previously [10]. Green channel images from an RGB stack were used for densitometric quantification of collagen content with ImageJ (NIH, Bethesda, MA). AGEs were assessed by immunohistochemical visualization, as described previously [31]. Briefly, sections were washed and incubated in primary antibody (1:200, GeneTex 20055) or negative control (2.5% horse serum, Vector Labs) overnight. AGEs were visualized using the appropriate secondary antibody and Vector Labs NovaRed (SK-4800) Peroxidase substrate kit. Three separate, blinded observers (DRM, YA, VRG) scored images on a zero to three scale (0 = absence of appreciable positive stain, 1 = minimal positive stain, 2 = appreciable positive stain, 3 = highly positive stain). Scores for each section were averaged across observers and normalized to negative control sections. Calcium deposition was investigated using Von Kossa staining, as previously described [32]. Briefly, sections were fixed with acetone (-20°C) for 10 min, washed 3 times with distilled water, incubated with 1% silver nitrate solution, and exposed to ultraviolet light for 20 min. After a final wash and removal of unreacted sliver with 5% sodium thiosulfate, sections were dehydrated with ethanol and xylene. Calcium particles were observed in visual fields at 10x magnification. The quantification of von Kossa staining was performed as described previously [33]. The surface of the aorta and calcification modules were manually measured with Image J. The calcified area was defined as calcified surface/tunica media (pixels/pixels).

### Statistics

Statistics were performed using SPSS (IBM, Chicago, IL). A 2- and 3-factor ANOVA was employed to evaluate differences between age (grouped at 3-month increments) and genotype (age X genotype), as well as between age, genotype, and sex (age X genotype X sex). Although there were differences in body mass and some tissue masses between male and female mice (data not shown), these data are not discussed because we observed no sex-related differences in our primary outcome, PWV. When a significant ANOVA was present, a least significant difference *t*-test was conducted to determine differences between values. Due to the number of comparisons, a Bonferroni correction was applied to within and between group comparisons for aortic PWV and heart rate data. Multiple linear regression was performed to model the relationships of genotype and/or sex with the age-related aortic stiffening. Statistical significance was set at P<0.05 for all analyses. Data are presented as mean±SEM.

### Ethical approval

All animal procedures conformed to the *Guide to the Care and Use of Laboratory Animals: Eighth Edition* [28] and were approved by the University of Utah and Veteran’s Affairs Medical Center-Salt Lake City Animal Care and Use Committees.
